# Factors impacting hospitalisation and related health service costs in cancer survivors in Australia: Results from a population data linkage study in Queensland (COS‐Q)

**DOI:** 10.1002/cam4.70201

**Published:** 2024-09-10

**Authors:** Katharina M. D. Merollini, Louisa G. Collins, Andrew T. Jones, Joanne F. Aitken, Michael G. Kimlin

**Affiliations:** ^1^ School of Health University of the Sunshine Coast Maroochydore Queensland Australia; ^2^ Sunshine Coast Health Institute Birtinya Queensland Australia; ^3^ Health Economics, Population Health Department QIMR Berghofer Medical Research Institute Brisbane Queensland Australia; ^4^ School of Nursing Queensland University of Technology Brisbane Queensland Australia; ^5^ Centre for Health Services Research, Faculty of Medicine University of Queensland Brisbane Queensland Australia; ^6^ Cancer Council Queensland Brisbane Queensland Australia; ^7^ School of Public Health University of Queensland Brisbane Queensland Australia; ^8^ Faculty of Health Sciences & Medicine Bond University Robina Queensland Australia

**Keywords:** Australia, cancer survivors, healthcare, hospital cost, risk factors, treatment

## Abstract

**Background:**

The global economic cost of cancer and the costs of ongoing care for survivors are increasing. Little is known about factors affecting hospitalisations and related costs for the growing number of cancer survivors. Our aim was to identify associated factors of cancer survivors admitted to hospital in the public system and their costs from a health services perspective.

**Methods:**

A population‐based, retrospective, data linkage study was conducted in Queensland (COS‐Q), Australia, including individuals diagnosed with a first primary cancer who incurred healthcare costs between 2013 and 2016. Generalised linear models were fitted to explore associations between socio‐demographic (age, sex, country of birth, marital status, occupation, geographic remoteness category and socio‐economic index) and clinical (cancer type, year of/time since diagnosis, vital status and care type) factors with mean annual hospital costs and mean episode costs.

**Results:**

Of the cohort (*N* = 230,380) 48.5% (*n* = 111,820) incurred hospitalisations in the public system (*n* = 682,483 admissions). Hospital costs were highest for individuals who died during the costing period (cost ratio ‘CR’: 1.79, *p* < 0.001) or living in very remote or remote location (CR: 1.71 and CR: 1.36, *p* < 0.001) or aged 0–24 years (CR: 1.63, p < 0.001). Episode costs were highest for individuals in rehabilitation or palliative care (CR: 2.94 and CR: 2.34, *p* < 0.001), or very remote location (CR: 2.10, *p* < 0.001). Higher contributors to overall hospital costs were ‘diseases and disorders of the digestive system’ (AU$661 m, 21% of admissions) and ‘neoplastic disorders’ (AU$554 m, 20% of admissions).

**Conclusions:**

We identified a range of factors associated with hospitalisation and higher hospital costs for cancer survivors, and our results clearly demonstrate very high public health costs of hospitalisation. There is a lack of obvious means to reduce these costs in the short or medium term which emphasises an increasing economic imperative to improving cancer prevention and investments in home‐ or community‐based patient support services.

## Graphical abstract

In this population‐based, retrospective data linkage study we investigate a range of contributing socio‐demographic and clinical factors for cancer survivors relating to hospital admissions in the public system and their costs in Queensland, Australia. The objectives of this work were to (a) understand differences in characteristics of cancer survivors with hospitalisation versus without hospitalisation, (b) explore the relationship between a range of clinical and socio‐demographic factors and hospitalisation costs on a population level from a health service perspective and (c) identify factors contributing to high hospitalisation costs.

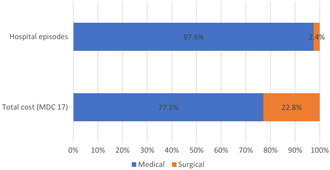

## 1 BACKGROUND

Cancer is a global public health priority, with 10 million cancer deaths reported in 2020 and mortality projections reaching 16 million individuals by 2040.^1^ Cancer incidence is expected to increase as many countries face growing and ageing populations whilst medical technologies continue to improve early detection and treatment of cancer. Individuals diagnosed with cancer in Australia have an overall relative 5‐year survival for all types of cancer combined of 70%.^2^

Globally, there was a prevalence of 43.8 million people alive in 2018 who were diagnosed with cancer in the last 5 years.^3^ This cohort is often referred to as ‘Cancer survivors’ and in the United States alone there are approximately 16.9 million individuals alive with a history of cancer,^4^ in Australia around 1.2 million.^5^ The economic and social impact of cancer survivorship is enormous on all levels of society, government systems, communities, individuals and their families. Health systems face ongoing challenges in providing adequate care for these patients, from diagnosis, through treatment, follow‐up, management of ongoing adverse effects on physical and psychological health and, for some, palliative care.

The future global economic cost of cancer is estimated to be $25.2 trillion international dollars from 2020 to 2050, with China, the United States and India expected to experience the highest absolute cost ($6.1, $5.3 and $1.4 trillion, respectively).^6^ Overall, high‐income countries were projected to have proportionally higher treatment costs contributing to total economic costs compared to low‐income countries, despite the latter accounting for 75% of death.^6^

It is important to understand health care utilisation from a provider perspective in order to identify factors impacting costs of care and to predict future resource allocation at a national and regional level. Recent publications have assessed factors influencing access to health services^7^, ^8^ and health care utilisation^9^, ^10^, ^11^, ^12^, ^13^ or the affordability of cancer care.^4^, ^14^, ^15^ But more research is needed to identify factors associated with hospitalisation and higher treatment costs relating to cancer to inform providers of future resource needs and prioritise programs that could reduce the burden on health services.

The Australian healthcare system is a mixed public and private system, built on the universal tax‐funded public health insurance scheme Medicare.^16^ It funds a range of primary and secondary care services and medicine at low‐cost or free of charge. Subsidised services include government‐owned inpatient and outpatient facilities, such as public hospitals, and other medical services listed on the Medicare Benefits Schedule, offered by qualified clinicians and allied healthcare providers.^17^ The private system offers alternative treatments and choice of provider for individuals willing to cover full fees or with private health insurance and typically results in higher patient out‐of‐pocket payments (gap payments) but shorter waiting times.^16^ Australian patients with cancer utilise a combination of services in the public and private systems, with a majority relying on the public health system for treatment.^18^ Health system expenditure relating to cancer is typically twice as high in public hospitals (46%) compared to private hospitals (22%), with the majority of all hospitalisations taking place in public hospitals.^19^, ^20^

In Australia, it is well‐established that hospital costs are the highest contributor to overall health service cost of cancer care^21^, ^22^, ^23^, ^24^ but it is not well‐understood if and how cancer survivors who are hospitalised differ from those who are not, and what drives overall hospital costs in this group. In particular, it is important to understand costs of patients treated in the public health system, rather than costs borne by private insurers and patients. The aim of this research was to characterise patients who are hospitalised in the public system and to identify factors related to these hospitalisations for cancer survivors in Queensland, Australia. The objectives were to (a) understand differences in characteristics of cancer survivors with hospitalisation versus without hospitalisation, (b) explore the relationship between a range of clinical and socio‐demographic factors and hospitalisation costs on a population level from a health service perspective and (c) identify factors associated with high hospitalisation costs.

## 2 METHODS

### 2.1 Study design

A population‐based, retrospective study was conducted, in which individual records of the Queensland Cancer Register were linked with administrative healthcare and cost data as part of the larger *Costs of Surviving Cancer in Queensland* (*COS‐Q*) study.^25^

### 2.2 Ethical considerations

Ethics approval was obtained from the Human Research Ethics Committee at the University of the Sunshine Coast (HREC Approval A/17/941) and the Australian Institute of Health and Welfare (HREC Approval EO2017/3/348). Project approval was obtained under Queensland's Public Health Act (2005) (grant RD007281).

### 2.3 Data sources

We used existing, linked administrative healthcare data for Queensland including data from 1997 to 2016 as outlined previously.^25^ These data include linked records for pharmaceuticals, medical and allied health services, hospitalisations, and emergency services as well as related costs and a range of socio‐demographic and clinical information.^23^ The data were routinely collected by Government agencies, mainly for reimbursement purposes, and were linked and de‐identified by the Statistical Services Branch, Queensland Health.^25^ For this study, we focussed on Queensland Cancer Register and hospitalisation records retrieved from the Queensland Hospital Admitted Patient Data Collection (QHAPDC).

### 2.4 Cohort selection

All individuals diagnosed with a first primary cancer between January 1997 and December 2015 and residing in Queensland were included in the COS‐Q linked dataset, as recorded in the Queensland Cancer Register (a state‐wide, legally mandated registry of all cancer diagnoses in the state of Queensland, excluding basal cell and squamous cell cancers of the skin). For this analysis, we selected individuals with a history of cancer with public hospital cost data incurred between January 2013 and December 2016 to focus on the most recent healthcare costs available, including various times of time since diagnosis (between 0 and 20 years prior). Hospital records contained any hospitalisation in the public setting, including treatment of any subsequent cancer diagnoses and non‐cancer related services.

### 2.5 Statistical analyses and variables of interest

Data cleaning and cost calculations have been described previously.^23^ In summary, we used a bottom‐up costing approach based on individual costs per hospital episode where total costs were defined as the product of direct and overhead costs. Student *t*‐tests (normally distributed variables) and Mann–Whitney U tests (non‐normally distributed variables) were conducted on the cancer cohort to assess differences between the with and without hospitalisation groups; Pearson's chi‐square tests were used to assess the associations between categorical cohort characteristics and hospitalisation record groups.

The dependent variables tested were all continuous, including mean annual hospital costs and mean hospital episode costs. Given the positive skewness and non‐negative values of healthcare costs, generalised linear models (GLM) with a log‐link function and gamma distribution were fitted to identify factors influencing hospital costs of cancer survivors.^26^ The explanatory variables used in the models included socio‐demographic factors (age, sex, country of birth, marital status, occupation, geographic remoteness category and socio‐economic index) and clinical factors (type of cancer by chance of relative 5‐year survival, year of/time since cancer diagnosis, vital status and care type). Geographic remoteness was measured using the Accessibility/Remoteness Index of Australia (ARIA) which is a measure of remoteness based on road distance to service centres; socio‐economic index was measured using the Socio‐Economic Indexes for Areas (SEIFA) summarising the relative socio‐economic characteristics of Australian communities, both measures were created by the Australian Bureau of Statistics.^27^, ^28^

Results of the GLM analyses were reported as cost ratios (CR) which reflect multiplicative differences between the mean of the reference group (referent) and the mean of group of interest. Relevant interaction terms were included in the model for applicable variables (age*occupation, ARIA*SEIFA status). Confidence intervals were reported using a 95% confidence level, and *p*‐values less than 0.05 were considered statistically significant.

Chance of relative 5‐year survival was defined using national and international criteria^29^, ^30^ as follows: low surviving cancers with a relative 5‐year survival of 0%–35% include brain, liver, pancreatic, lung, oesophageal and stomach cancer, as well as cancer with unknown primary site; medium surviving cancers are defined by having a relative 5‐year survival of 36%–69% and include myeloma, leukaemia, bladder, ovarian and kidney cancer, and high surviving cancers are defined by having a relative 5‐year survival of 70%–100% and include: Non‐Hodgkin's Lymphoma, head and neck, colorectal, breast, prostate, cervical, uterine and thyroid cancer as well as melanoma.

Hospital episode costs were also analysed using Separation statistics: Major Diagnostic Categories (MDC) and Australian‐Refined Diagnosis Related Groups (AR‐DRGs) (version 5.0 to 7.0, from 2013 to 2016), as published by the Australian Institute of Health and Welfare based on classifications from the Independent Hospital Pricing Authority (IHPA).^31^ All analyses were conducted using IBM SPSS Statistics version 27. Costs were converted to March 2024 Australian dollars to account for inflation over time using the Australian Consumer Price Index (Health).^32^

## 3 RESULTS

### 3.1 Cohort of interest

Cohort selection from the larger COS‐Q linked dataset^25^ is illustrated in Figure S1. From the initial patient cohort of 365,443 individuals diagnosed with a first primary cancer between 1997 and 2015, we selected a cohort of 230,380 individuals who were both still alive in 2013 and incurred healthcare costs during the study period (2013–2016) as recorded in one or more of the linked datasets (pharmaceuticals, public hospitalisations, emergency admissions, allied health and medical services). We were further able to identify 111,820 individuals within this cohort with hospitalisation records in the public setting (study cohort for objectives b and c relating to hospital costs), leaving 118,560 without hospitalisation records (relevant only for objective a to understand differences between characteristics of cancer survivors with hospitalisation vs. no hospitalisation).

### 3.2 Differences in cancer survivors with versus without hospitalisation

#### 3.2.1 Socio‐demographic and clinical characteristics

The characteristics of the cohort of 230,380 individuals with cost data^25^ is summarised in Table 1 below, stratified by hospitalisation to capture differences between these groups in terms of sociodemographic and clinical factors. Just under half of the cohort (48.5%) experienced hospital admissions in the public setting. Compared to the group without hospitalisation, the hospitalised group was statistically significantly different for all variables described below (*p*‐values <0.05).

**TABLE 1:** Descriptive characteristics of cancer cohort (*N* = 230,380) stratified by hospitalisation between 2013 and 2016.

	Hospital admissions		No hospital admissions		*p*‐Value
	*N*	(%)	*N*	(%)	
Total	111,820	48.5	118,560	51.5	
Socio‐demographic factors					
Sex^a^					
Male	61,225	54.8	57,912	51.5	<0.001
Female	50,595	45.2	54,467	48.5	
Age at diagnosis in years					
0–24	2270	2.0	3018	2.5	<0.001
25–49	16,658	14.9	26,844	22.6	
50–64	36,202	32.4	45,798	38.6	
65–74	32,191	28.8	27,788	23.4	
75+	24,499	21.9	15,112	12.7	
Mean age (SD)	62.8 (15.2)		58.4 (15.2)		<0.001
Country of birth					
Australia	83,833	75.0	93,161	78.6	<0.001
Other	27,987	25.0	25,399	21.4	
Marital status					
Married/de facto	68,907	61.6	83,520	70.4	<0.001
Widowed/divorced/separated	31,482	28.2	20,279	17.1	
Never married	11,267	10.1	11,254	9.5	
Unknown	164	0.1	3507	3.0	
Occupation					
Working	49,579	44.3	48,809	41.2	<0.001
Retired/not in work/home duties	36,197	32.4	23,049	19.4	
Children/students	1342	1.2	1505	1.3	
Inadequately described	24,702	22.1	45,197	38.1	
Socio‐Economic Indexes for Areas (SEIFA)					
1–2 (Most disadvantaged)	14,649	13.1	9550	8.1	<0.001
3–4	28,554	25.5	21,098	17.8	
5–6	28,179	25.2	26,534	22.4	
7–8	21,395	19.1	26,923	22.7	
9–10 (Most advantaged)	6847	6.1	11,126	9.4	
Unknown	12,196	10.9	23,329	19.7	
Accessibility and Remoteness Index of Australia (ARIA)					
Major cities	58,339	52.2	68,724	58.0	<0.001
Inner regional	30,936	27.7	26,358	22.2	
Outer regional	19,578	17.5	13,544	11.4	
Remote	1526	1.4	918	0.8	
Very remote	1211	1.1	610	0.5	
Unknown	230	0.2	8406	7.1	
Clinical factors					
Year of cancer diagnosis					
1997–2001	12,054	10.8	16,442	13.9	<0.001
2002–2006	18,902	16.9	26,135	22.0	
2007–2011	30,690	27.4	38,654	32.6	
2012–2015	50,174	44.9	37,329	31.5	
Type of cancer					
Low surviving	13,382	12.0	5822	4.9	<0.001
Medium surviving	11,241	10.1	8855	7.5	
High surviving	78,070	69.8	95,244	80.3	
Other/mixed/unknown	9127	8.2	8639	7.3	
Vital status					
Alive in 2016	92,125	82.4	111,370	93.9	<0.001
Died prior to 2016	19,695	17.6	7190	6.1	
Age at death in years, mean (SD)	73.5 (13.5)		77.9 (13.0)		0.014
Years lived after diagnosis, mean (SD)	3.9 (4.5)		4.5 (5.0)		0.004

^a^ Due to data linkage, there were *n* = 6181 (2.7%) individuals with missing values for ‘sex’ in the group without hospitalisations.

There was a slightly higher representation of males in the hospital group compared to the group without hospitalisation (54.8% vs. 51.5%). The mean age at diagnosis was 4.5 years higher in the hospital group (62.8 vs. 58.4 years) due to a lower proportion of individuals diagnosed at 25–49 years (14.9% vs. 22.6%) and a higher proportion diagnosed at 75+ years (21.9% vs. 12.7%). The hospital group had a higher proportion of individuals born outside Australia (25.0% vs. 21.4%), fewer married or in a de facto relationship (61.6% vs. 70.4%), more who were widowed, divorced or separated (28.2% vs. 17.1%) and more not currently working (32.4% vs. 19.4%) compared to the group without hospital admissions. Another noticeable difference was that the hospital group included more socio‐economically disadvantaged persons (SEIFA 1–4: 38.6% vs. 25.9%) and nearly twice as many from very remote and remote geographic locations (2.5% vs. 1.3%).

In terms of clinical characteristics, a larger proportion of the hospital group was diagnosed during the study costing period between 2012 and 2015 (44.9% vs. 31.5%), were diagnosed with a cancer type with low (12% vs. 4.9%) or medium (10.1% vs. 7.5%) chance of 5‐year survival, died prior to 2016 (17.6% vs. 6.1%), and with a lower age at death (73.5 vs. 77.9 years) and lower survival since their initial diagnosis (3.9 vs. 4.5 years).

#### 3.2.2 Healthcare utilisation and costs

Overall health service use and mean annual healthcare cost per person per year were compared between the groups (Table S1). Of the 111,820 individuals with hospitalisations during the study period, the majority experienced emergency presentations (80.9%) whereas of the 118,560 individuals without hospital admission this was the case for only a minority (16.8%). Utilisation of medical and allied health services (96.9% vs. 97.5%) and prescribed pharmaceuticals (97.9% vs. 97.5%) was similar in both groups.

Mean annual healthcare costs incurred per cancer survivor and type of health service used are illustrated in Figure S2 and numerically in Table S1. The total mean annual healthcare costs were 4.4 times higher for individuals who experienced hospital admissions during the study period (between 2013 and 2016) compared to those who did not (AU$37,103 vs. AU$8350, *p* < 0.001). Mean annual costs per person for each type of health service were significantly higher in the group with hospital admissions, including emergency presentations (+AU$1343), medical and allied health services (+AU$486) and pharmaceuticals (+AU$2367); this group also had mean annual costs for hospitalisation of AU$24,281, which contributed the most to the overall cost difference of AU$28,753.

### 3.3 Socio‐demographic and clinical factors associated with annual hospital costs

Results of the GLM analysis for the mean annual hospital costs of cancer survivors were reported as cost ratios (CR) (Table S2). All tested variables were independently associated with the mean annual hospital costs at a significance level of 0.05 including socio‐demographic factors sex, age at diagnosis, country of birth, marital status, occupation, socio‐economic index and geographic remoteness category, as well as clinical factors year of cancer diagnosis, type of cancer by relative 5‐year survival (low, medium and high) and vital status.

Factors with slightly but statistically significantly increased relative risk of higher mean annual hospitalisation costs (4%–17% increased) included being ‘widowed, divorced or separated’ (CR 1.17, *p* < 0.001, compared to married/de facto), cancer with medium change of relative 5‐year survival (CR 1.14, *p* < 0.001, compared to low surviving cancer), being male (CR 1.14, *p* < 0.001, compared to female), aged 0–24 years (CR 1.63, *p* < 0.001, compared to 75+ years), outer regional location (CR 1.06, *p* < 0.001, compared to major cities), socio‐economically most disadvantaged (CR 1.09, *p* < 0.001, compared to most advantaged) or born outside Australia (CR 1.06, *p* < 0.001, compared to in Australia).

Factors associated with much higher annual hospitalisation costs (24%–80% increases) included having died during the study period (CR 1.79, *p* < 0.001), living in a very remote (CR 1.71, *p* < 0.001) or remote location (CR 1.36, *p* < 0.001, compared to major city), age 0–24 years (CR 1.63, *p* < 0.001), having occupation ‘children/students’ (CR 1.39, *p* < 0.001, compared to ‘working’), having a recent cancer diagnosis (CR 1.35, *p* < 0.001, 2012–2015 compared to 1997–2001), and never being married (CR 1.24, *p* < 0.001, compared to married/de facto).

### 3.4 Factors associated with higher hospital episode costs

#### 3.4.1 Most common reasons for hospital admissions and related costs

The *n* = 111,820 individuals with hospitalisation had a total of *n* = 682,483 hospital episodes recorded during the study period. The mean length of stay (LoS) was 3.0 days (SD 6.7) and the mean costs per hospital episode of AU$7168 (SD 12,929). Reasons for hospital admission can broadly be categorised using Major Diagnostic Categories (MDC) (see Table S3) and most common reasons included ‘11: Diseases and disorders of the kidney and urinary tract’ with 20.9% of all hospital episodes, closely followed by ‘17: Neoplastic disorders’ with 19.7% of hospital episodes. Total cohort costs by MDC were highest for ‘6: Diseases and disorders of the digestive system’ with AU$661.1 million (13.5% of total cost), followed by ‘17: Neoplastic disorders’ with AU$553.8 million (11.3% of total cost) and ‘4: Diseases and disorders of the respiratory system’ with AU$471.3 million (9.6% of total cost). MDCs with the longest mean length of stay in hospital were ‘19: Mental diseases and disorders’ (8.8 days, SD: 22.6), ‘1: Diseases and disorders of the nervous system’ (5.4 days, SD: 11.3) and ‘8: Diseases and disorders of the musculoskeletal system and connective tissue’ (5.3 days, SD: 8.3).

**FIGURE 1:**
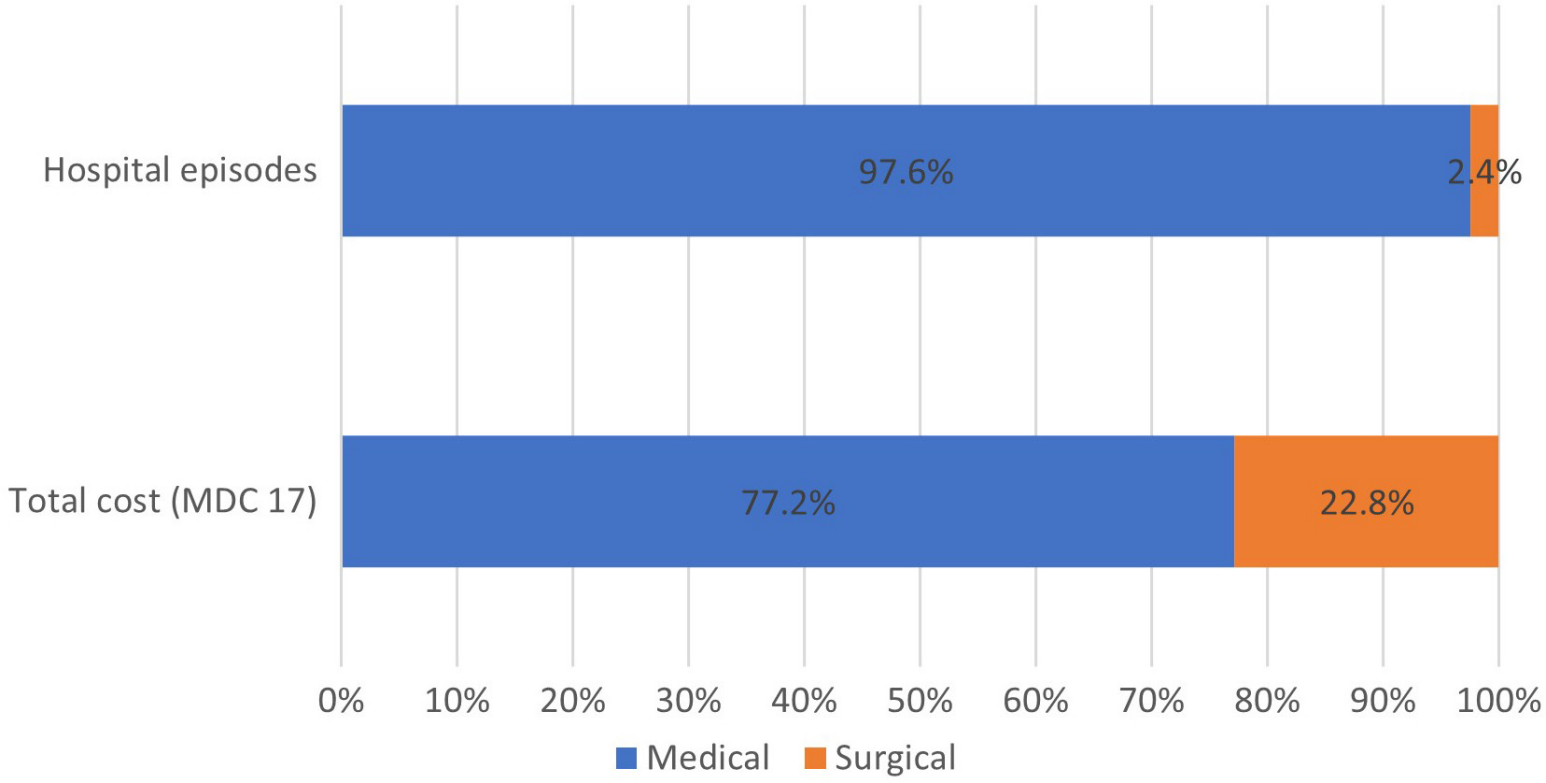
Overview of ‘MDC 17 Neoplastic disorders’ frequency and health service cost by type of procedure for *n* = 134,427 hospital episodes (2013–2016).

Hospital episodes directly related to cancer treatment with MDC category 17 ‘Neoplastic disorders’ are summarised in Table S4 with description of AR‐DRG codes, costs and length of stay. From a total of 134,427 hospital episodes, 97.6% of episodes were due to medical interventions which incurred 77.2% (AU$427 million) of total MDC 17 costs, whereas only 2.4% of hospital episodes were for surgical procedures which incurred 22.8% of costs (AU$126 million) (Figure 1).

**FIGURE 2:**
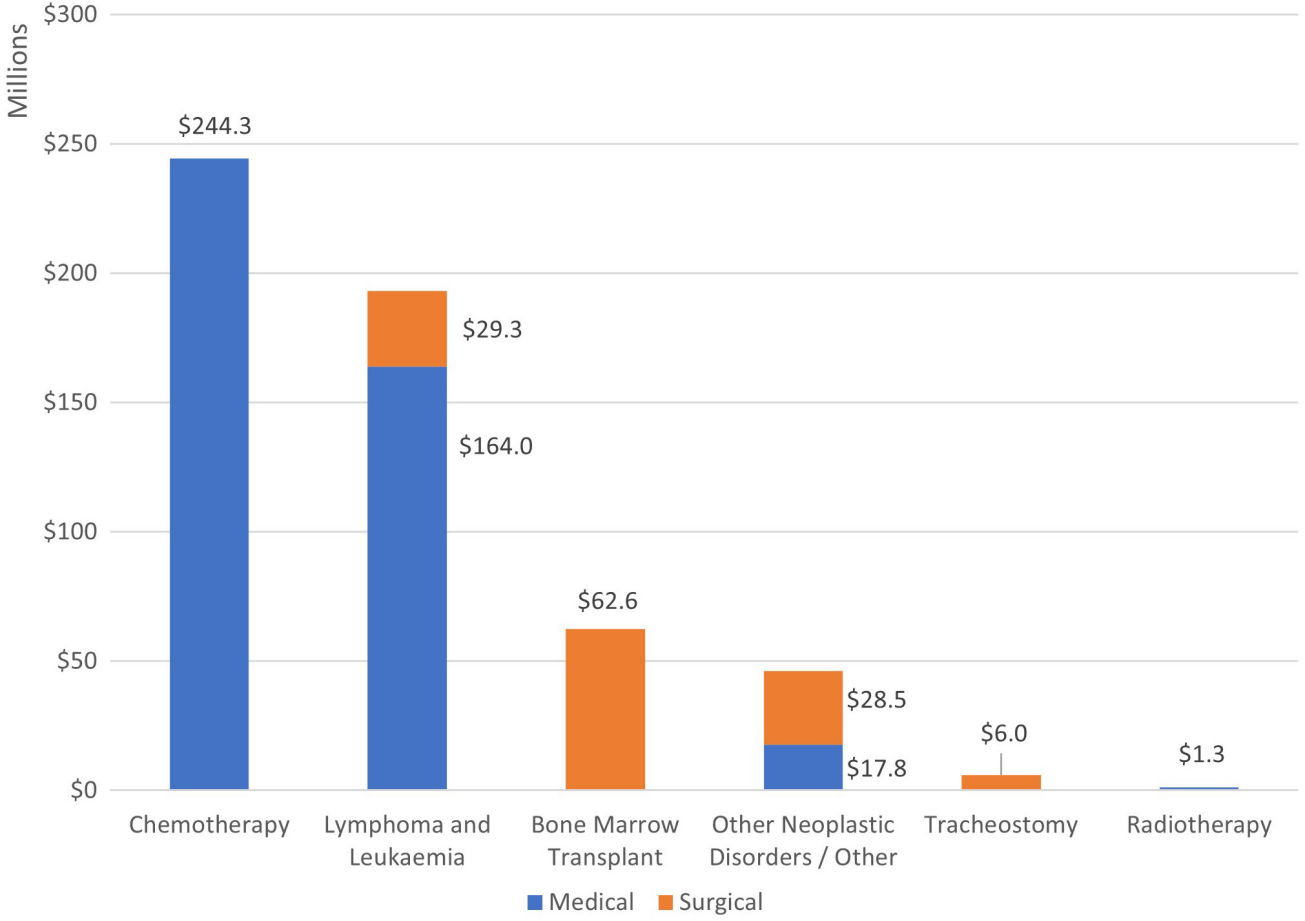
Overview of cumulative costs of ‘MDC 17 Neoplastic disorders’ by treatment for *n* = 134,427 hospital episodes (2013–2016).

Highest incidence and highest total costs during the study period were incurred by chemotherapy hospital episodes (83.2% of MDC 17 episodes) resulting in AU$244.3 million health service costs (44% of MDC 17 cost), as illustrated in Figure 2. Lymphoma and Leukaemia hospital admissions accounted for 13.6% of MDC 17 admissions and cost a total of AU$193.3 million (AU$164.0 million medical, AU$29.3 million surgical), whereas bone marrow transplants only accounted for 0.5% of episodes but 11% of costs with AU$62.6 million total costs. Tracheostomy and radiotherapy had the lowest total costs and lowest number of hospital admissions (AU$6.0 million and AU$1.3 million, respectively). Longest mean length of hospital stay per episode of care were for tracheostomy and bone marrow transplants (26.1 and 24.9 days, respectively). ‘Other neoplastic disorders/other’ accounted for AU$28.5 million due to surgical and AU$17.8 million due to medical hospital treatments, with a total of 2.2% of MDC17 admissions.

#### 3.4.2 Socio‐demographic and clinical factors impacting hospital episode costs

Results from the GLM are reported as cost ratios (CR) (Table S5). Socio‐demographic factors associated with higher hospital episode costs were living in very remote location (CR 2.10, *p* < 0.001, compared to major cities), being male (CR 1.08, *p* < 0.001), widowed/divorced/separated (CR 1.07, *p* < 0.001), never married (CR 1.06, *p* < 0.001, compared to married) aged 0–24 years (CR 1.05, *p* = 0.05, compared to 75+ years) or born outside Australia (CR 1.01, *p* < 0.05, compared to in Australia). Individuals from most advantaged socio‐economic backgrounds (SEIFA 7–10) had higher mean costs per hospitalisation compared to most disadvantaged groups (CR 1.07, *p* < 0.001). Based on individual hospital episode costs, children/students had significantly lower costs (CR 0.85, *p* < 0.001) compared to working individuals. Clinical factors associated with higher hospital episode costs were hospital care types ‘rehabilitation’ (CR 2.94, *p* < 0.001) or ‘palliative care’ (CR 2.34, *p* < 0.001, compared to acute care), when an individual died during a hospital episode (CR 1.61, *p* < 0.001) or was hospitalised during the first year post cancer diagnosis (CR 1.38, *p* < 0.001), 2–4 years ago (CR 1.09, *p* < 0.001) or 5–9 years ago (CR 1.04, *p* < 0.001) compared to 15–20 years ago. The care type summarised as ‘other’ due to small numbers, showed nearly 4‐times higher costs (CR: 3.91, *p* < 0.001) compared to ‘acute care’ (AU$6442, SD: 11,953): ‘Maintenance care’ (0.7% of episodes, AU$28,061, SD:38,728), ‘Geriatric Evaluation and Maintenance’ (0.5%, AU$25,572, SD:20,026), ‘Mental health care’ (0.2%, AU$17,953, SD:31,287), ‘Boarder’ (0.1%, AU$2368, SD: 27,891) and ‘Newborn’ (0.0%, AU$36,920, SD: 50,051). Individuals with cancer type with low chance of 5‐year survival had higher mean episode costs compared to medium surviving cancer (CR 0.89, *p* < 0.001) but lower costs compared to high surviving cancers (CR 1.04, *p* < 0.001).

#### 3.4.3 Costs younger age group: 0–24 years

Mean costs of palliative care hospital admissions (*n* = 73, 0.4%) were highest in the 0–24 year age group (*n* = 19,290) with a mean episode cost of AU$24,204 (SD: 25,295) and mean length of stay of 6 days (SD: 6) compared to acute care admissions in this group (*n* = 19,014, 98.6%) with mean costs of AU$7875 (SD: 20,105) and mean LoS of 2 days (SD: 5). Highest incidence (*n* = 8399, 43.5%) and highest overall costs (AU$59.7 million) in this age group were for leukaemia which had a mean cost per episode of care of AU$7107 (SD: 22,055). Highest mean costs per hospital admission were for lung cancer (AU$17,641.1, SD: 37,278), Non‐Hodgkin lymphoma (AU$14,361, SD: 26,345) and stomach cancer (AU$12,227, SD: 16,878). Compared to the overall cohort, the 0–24 year age group had a higher proportion of ‘MDC 17: Neoplastic Disorders’ admissions (51.1% vs. 11.3%) accounting for 37.6% (AU$57.8 m) of total costs in this group (AU$154.0 m), followed by ‘MDC 16 Diseases and Disorders of the Blood and Blood Forming Organs and Immunological Disorders’ with 8.2% of admissions (total cost of AU$14.3 m) and ‘MDC 8: Diseases and disorders of the musculoskeletal system and connective tissue’ with 6% of admissions (total cost of AU$15.7 m). The most common treatment codes within ‘MDC 17: Neoplastic Disorders’ were for chemotherapy (79.2%, total cost AU$12.9 m), acute leukaemia (7.0%, total cost AU$4.6 m) and radiotherapy (5.3%, total cost AU$1.3 m).

### 3.5 Summary of factors associated with higher hospital costs

**FIGURE 3:**
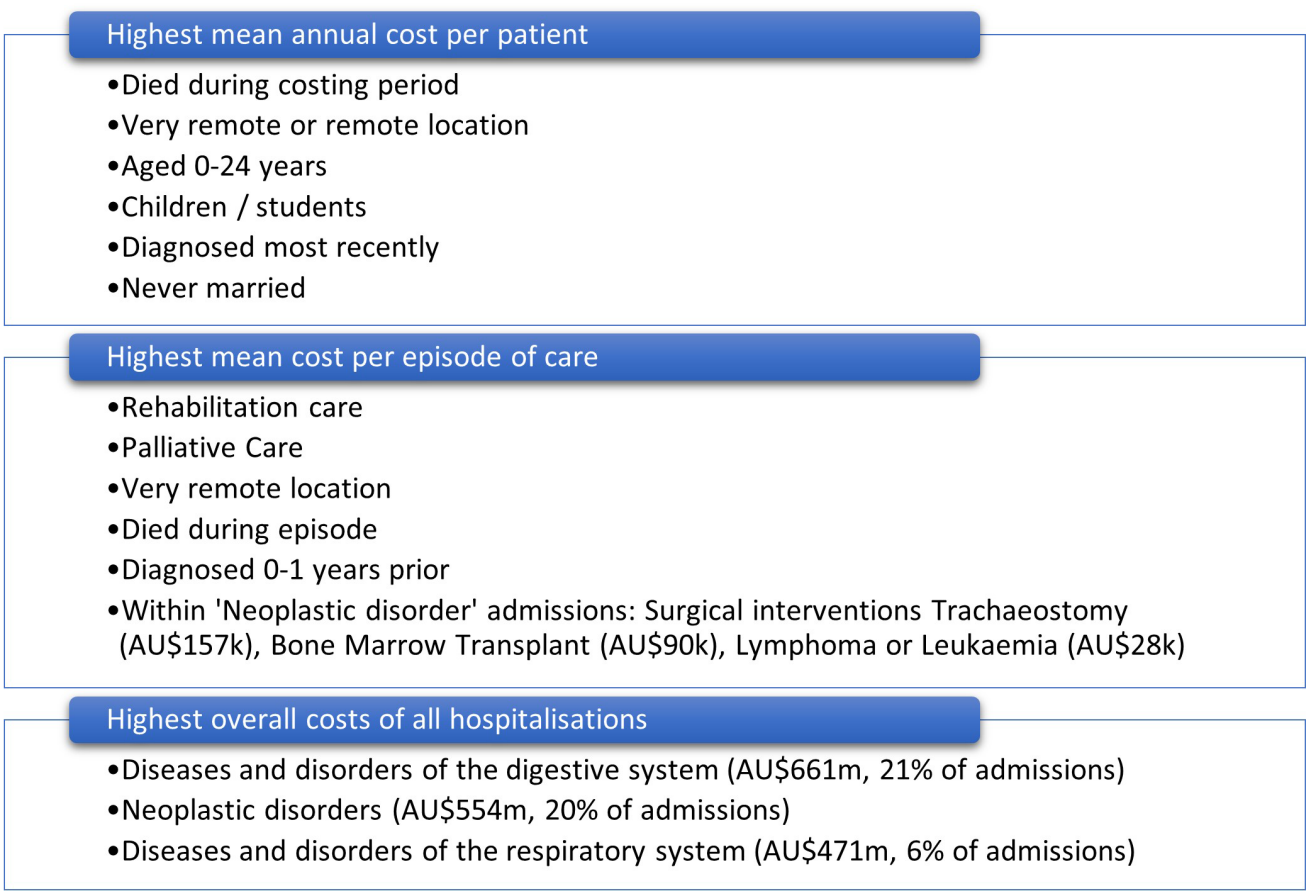
Summary of factors associated with higher hospital costs.

Socio‐economic and clinical factors associated with higher mean annual hospital costs per person, higher mean cost per episode of care and highest overall costs of hospitalisation by major diagnostic category are summarised in Figure 3, based on findings from different study components described previously.

## 4 DISCUSSION

### 4.1 Interpretation of results

Our data showed that in our cohort of long‐term cancer survivors, just under half (48.5%) had at least one public hospital admission, resulting in a mean cost difference of AU$28,753, compared to individuals without. This difference is explained by high mean annual hospitalisation costs (AU$24,281, SD: 27,542) and more emergency presentations in the hospital group (81% vs. 17%). Other characteristics of individuals with hospitalisations included older age at time of diagnosis (63 vs. 58 years), higher socio‐economically disadvantage (39% vs. 26%), not as likely to be in a married/de facto relationship (62% vs. 70%), more likely to be divorced (28% vs. 17%), low surviving cancer (12% vs. 5%), death (17 vs. 6%), and residing in remote or very remote locations (2.5% vs. 1.3%). It should be noted that our data did not include private hospitalisations and individuals with private health insurance or personal funds to cover private providers may have had hospitalisations outside the public system. Our finding of a higher proportion of low socio‐economic backgrounds in the hospital group may be explained by the fact that individuals from higher socio‐economic backgrounds may also use other private hospital services.

The strongest predictor of higher annual hospital costs per person in our data was death during the study period, that is, between 2013 and 2015 (as they still had to incur some healthcare costs between 2013 and 2016 to be eligible for this analysis) which increased costs by 80% (CR = 1.79). This finding aligns with well‐established research that has shown that the last year of life is associated with higher healthcare costs than average.^24^, ^33^, ^34^ This finding is also in line with higher mean hospital costs if patients were diagnosed between 2012 and 2015 (CR = 1.35) which can be explained by the majority of the patients their initial cancer treatment being captured in our cost data ranging from 2013 to 2016 (44.9% in hospital group) as well as a proportion of patients dying during these years (17.6% in hospital group). Furthermore, living in a very remote or remote location, aged 0–24 years, occupation child/student, diagnosed most recently, or never married were factors associated with 24%–71% higher costs. Other factors increasing costs by 4%–17% included marital status ‘widowed/divorced/separated’, being male, cancer with medium chance of relative 5‐year survival, living in outer regional location, socially disadvantaged and born outside Australia. Higher costs for individuals without partner (i.e. never married) may stay in hospital longer due to a lack of social support at home, which in turn would increase their hospital costs. It should also be noted that some cost ratios, although statistically significant due to our large sample size, may not be clinically meaningful, and may be a testament to the public system meeting population needs.

When predicting high episode costs, some of these were similar to the factors identified for mean annual cost of hospitalisation above, such as having died during an episode of care, diagnosed recently (within last year) or living in very remote location compared to major city location. Furthermore, mean episode costs were the highest for patients in rehabilitation and palliative care compared to acute care, which also had longer length of hospital stay (11.0 and 8.9, compared to 2.4 days in acute care) which is a direct proxy for healthcare costs.

In terms of diagnosis and treatment codes, the highest contributors to overall costs were ‘diseases and disorders of the digestive system’ (AU$661 m, 21% of admissions) and ‘neoplastic disorders’ (AU$554 m, 20% of admissions). As the latter were cancer‐specific we focussed on investigating these by sub‐codes and found that surgical procedures incurred higher mean costs (ranging from AU$19,512—AU$156,897) compared to medical interventions (AU$2186—AU$18,756) but medical procedures were responsible for overall higher proportion of costs due to a higher incidence (77.2% vs. 22.8%). The most common type of medical procedures within ‘neoplastic disorders’ was ‘chemotherapy’ which accounted for 83% of these hospital episodes and 44% of costs (relating to ‘neoplastic disorders’ costs). This finding is plausible as a large proportion of individuals diagnosed with cancer will undergo chemotherapy. A surprising finding was that only 0.5% of hospital admissions in relating to ‘neoplastic disorders’ were due to radiotherapy. This can be partially explained by a large proportion of Australian patients treated for cancer undergoing radiotherapy outside the hospital setting, such as in privately run treatment clinics as radiation oncology is funded by the government (via Medicare with 75%–85% coverage of cost).^5^, ^35^, ^36^ Highest cost ‘neoplastic disorders’ were surgical interventions which are by nature more resource intensive, including tracheostomy (AU$157 k/episode), bone marrow transplants (AU$90 k/episode) and for lymphoma or leukaemia (AU$28 k/episode). Some of these also had the longest length of stay, which again was a proxy for higher hospital episode costs (tracheostomy: mean 26.1 days; bone marrow transplant: mean 24.9 days; surgical lymphoma/leukaemia: mean 8 days in hospital).

Children/students and young people aged 0–24 years were identified as factors in increasing costs by up to 63%. This may be explained by the fact that our data included palliative care admissions (*N* = 73) in this younger age group, which were also identified as high‐cost factor with higher mean episode cost (AU$24,204, SD: 25,295) and longer mean length of hospital stay (6 days, SD:6) compared to otherwise low costs for hospital episodes in this age group (AU$7875, SD: 20,610) and relatively short length of stay (2 days, SD:5). Highest overall costs were due to leukaemia which accounted for AU$4.6 million of total episode costs and may have driven mean costs up. The cost driver marital status ‘never married’ may also be indirectly linked to children/students and young people aged 0–24 years which are in most cases not married, although the models accounted for correlations between age and occupation status.

Interestingly, cancer survivors with medium chance of relative 5‐year survival had higher mean annual healthcare costs compared to cancer survivors with low chance of relative 5‐year survival. This might be partially explained by the fact that myeloma and leukaemia are both classed as medium‐surviving cancers in this analysis. Both of these cancer types are known to incur much higher‐than‐average costs and require ongoing treatment to combat adverse effects.^23^, ^37^ Another explanation could be that individuals with low surviving cancer are not accessing care for as long as their counterparts.

Recently published work on healthcare costs of breast cancer survivors in the United States noted that these costs are often driven by a small group of high‐cost individuals and may include unnecessary or irrelevant services but may also be due to underlying demographic and social‐economic factors.^38^ They found that the use of chemotherapy drugs and the interval of days without chemotherapy had the biggest impact on these costs but also receipt of surgery or radiation and insurance type were major cost drivers.^38^ These findings are similar in the sense that hospital‐based services and in particular chemotherapy were the highest cost contributors within neoplastic disorder hospital admissions although our data did not include many radiotherapy admissions. In the Australian context, a study on lung cancer in contrast to our results identified higher health system costs for individuals diagnosed at between 45 and 69 years and found no evidence of differences in costs by sex or year of diagnosis.^39^

Overall, it is expected that hospital costs will increase over time. Even in an unlikely scenario where mean costs per patient remained the same, there would be higher costs to the public system due to the sheer increase in the number of cancer survivors alone. As there are no means to reduce hospital costs in the short‐ to medium‐term, it is imperative to focus on cancer prevention from an economic perspective alone.

### 4.2 Strengths and limitations

Limitations of this work include that our data were based on routinely collected administrative data, which was not designed for research, and it mainly included public healthcare cost data rather than private health service use, with exception to private services subsidised by Medicare under the Medicare Benefit Schedule for overall health service costs. We also did not have information on cancer stage at diagnosis as this is not recorded in the Queensland Cancer Register. This work only focussed on direct healthcare costs from a health services perspective and did not include societal costs, such as productivity losses, or indirect costs to patients, such as out‐of‐pocket payments for over‐the‐counter medication or travel costs to and from medical appointments. Furthermore, given the study design it was not possible to distinguish costs related to cancer and/or co‐morbidities from overall healthcare costs and as in any population dataset there was large heterogeneity within types and length of hospital admissions. Further correlations or other confounding factors between variables may exist and are known to be unavoidable but are currently unknown. Strengths of this work are the use of highly reliable population data for Queensland, Australia, spanning over two decades and including linkage of cancer diagnoses (first primary malignancy) using the population‐based Queensland Cancer Register. We captured a large number of important socio‐demographic and clinical factors to explore their impact on hospital costs (annual per patient and episode costs) and developed a statistical model that was able to show the relationship between these variables and overall annual hospital costs incurred on a patient level as well as episode costs. Costs presented are a snapshot of annual costs incurred at different stages post cancer diagnosis and were not meant to capture lifetime costs per patient. Our results are robust, identified groups with higher healthcare needs and inform future healthcare allocation.

### 4.3 Future research

The exploration of models including more dependent variables, such as cancer stage, co‐morbid conditions and general health status could be pursued. Research in other settings could add to this evidence presented here and potentially confirm the generalisability of findings to other settings. There is also a need to explore tailored surveillance programs for subgroups of individuals at risk of incurring high healthcare costs to allow early intervention and to reduce costly hospitalisations, for example by shifting some of these costs to home‐based or community support services, such as for palliative care and remote locations.

## 5 CONCLUSIONS

This research has identified a range of socio‐demographic and clinical factors associated with hospitalisations and high hospital costs in the public hospital system. Our findings demonstrate the significant public health costs associated with the growing number of cancer survivors and stress the importance of reducing hospitalisation rates where possible, such as by investing in home‐ or community‐based patient support services and early intervention to reduce some of these healthcare costs in the long term. There is a lack of obvious means to reduce these costs in the short or medium term which emphasises an increasing economic imperative to improve cancer prevention.

## AUTHOR CONTRIBUTIONS

**Katharina M. D. Merollini:** Conceptualization (equal); data curation (lead); formal analysis (lead); funding acquisition (lead); methodology (equal); project administration (lead); resources (lead); software (lead); writing – original draft (lead); writing – review and editing (equal). **Louisa G. Collins:** Conceptualization (equal); methodology (equal); supervision (equal); writing – review and editing (equal). **Andrew T. Jones:** Formal analysis (supporting); methodology (supporting); software (supporting); validation (supporting); writing – review and editing (equal). **Joanne F. Aitken:** Conceptualization (equal); methodology (equal); supervision (equal); writing – review and editing (equal). **Michael G. Kimlin:** Conceptualization (equal); funding acquisition (supporting); methodology (equal); supervision (equal); writing – review and editing (equal).

## FUNDING INFORMATION

This research received no specific grant from any funding agency in the public, commercial or not‐for‐profit sectors. KM used her institution fellowship funds to cover data linkage and data storage fees.

## CONFLICT OF INTEREST STATEMENT

The authors have no relevant financial or non‐financial interests to disclose.

## ETHICS STATEMENT

Ethics approval has been obtained from the University of the Sunshine Coast Human Research Ethics Committee (Ethics Approval A/17/941) and from the Australian Institute of Health and Welfare (AIHW) Human Research Ethics Committee (EO2017/3/348).

## CONSENT TO PARTICIPATE

Approval for Queensland data extraction and linkage, including a waiver of consent has been sought from Queensland Health under the Public Health Act from 2005 and was granted by the Director‐General (grant RD007281).

## Supporting information


Figures S1–S2.

Tables S1–S4.

## Data Availability

The data that support the findings of this study are available from the data custodians of each of the linked datasets, but restrictions apply to the availability of these data, which were used under licence for the current study, and so are not publicly available. Aggregated data used for this manuscript are however available from the authors upon reasonable request.
