# Supplementary material for: Factors impacting hospitalisation and related health service costs in cancer survivors in Australia: Results from a population data linkage study in Queensland (COS‐Q)
Source: Cancer Med. 2024 Sep 10;13(17):e70201. doi: 10.1002/cam4.70201 (PMC11386302; doi:10.1002/cam4.70201)
Supplement: Supplementary file 1 — Figures S1–S2. [file CAM4-13-e70201-s002.docx]

**Supplementary Figures** for article: Factors impacting hospitalisation and related health service costs in cancer survivors in Australia: results from a population data linkage study in Queensland (COS-Q)

Supplemen

Supplementary Figure S1: Flowchart of Included Individuals from COS-Q linked dataset

**
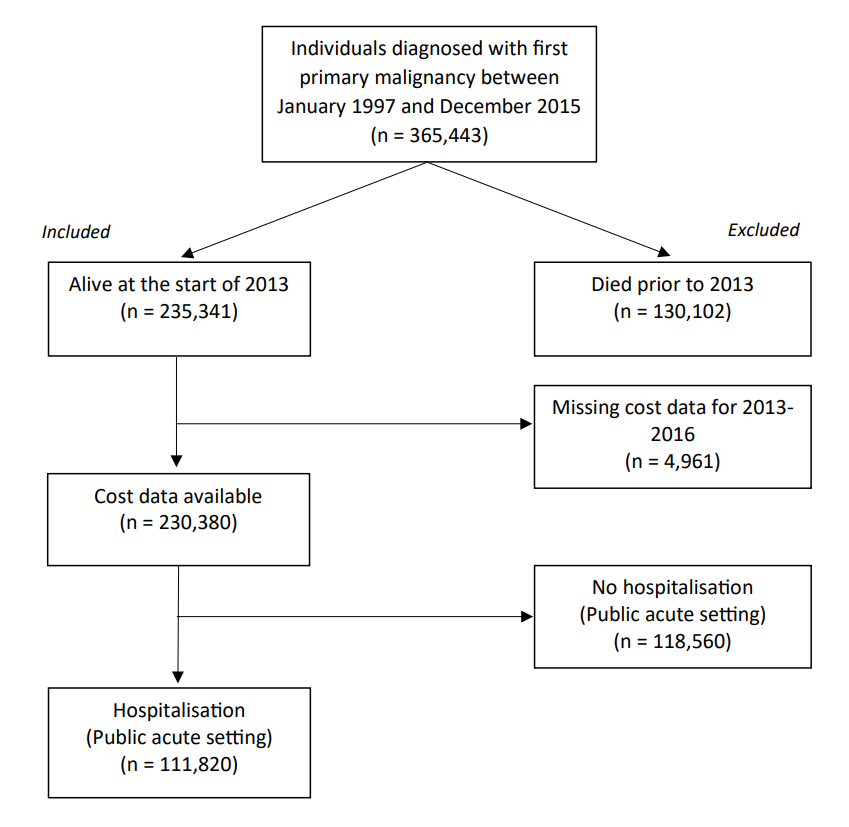
**

Supplementary Figure S2: Mean annual healthcare costs per person in AU$ by type of health service and hospitalisation group (N=230,380)
