# Supplementary material for: Factors impacting hospitalisation and related health service costs in cancer survivors in Australia: Results from a population data linkage study in Queensland (COS‐Q)
Source: Cancer Med. 2024 Sep 10;13(17):e70201. doi: 10.1002/cam4.70201 (PMC11386302; doi:10.1002/cam4.70201)
Supplement: Supplementary file 2 — Tables S1–S4. [file CAM4-13-e70201-s001.docx]

**Supplementary Tables** for article: Factors impacting hospitalisation and related health service costs in cancer survivors in Australia: results from a population data linkage study in Queensland (COS-Q)

Supplementary Table S1: Health service utilisation and cost comparison for N= 230,380 cancer survivors with vs without hospitalisation (2013 – 2016)

|  | **Hospital admissions** | | | | **No hospital admissions** | | | |  |  |
| --- | --- | --- | --- | --- | --- | --- | --- | --- | --- | --- |
|  |  |  |  |  |  |  |  |  |  |  |
| **Type of health service** | N | (%) | Mean annual cost per person in AU$ (SD) | Median annual cost per person in AU$ (IQR) | N | (%) | Mean annual cost per person in AU$ (SD) | Median annual cost per person in AU$ (IQR) | ∆ Cost difference in mean costs | P-value |
| Mean total healthcare cost | 111,820 | 100 | $ 37,103 (31,246) | $ 22,930 (24,696) | 118,560 | 100 | $8,350 (9,672) | $4,259 (4,678) | $28,753 | <0.001 |
| Hospitalisation | 111,820 | 100 | $ 24,281 (27,542) | $ 11,540 (18,570) | 0 | 0 | $0 | $0 | $24,281 | <0.001 |
| Emergency presentation | 90,490 | 80.9 | $ 2,308 (1,521) | $ 1,714 (1,210) | 19,930 | 16.8 | $964 (552) | $816 (451) | $1,343 | <0.001 |
| Medical & allied health services | 108,317 | 96.9 | $ 5,645 (4,365) | $ 3,898 (3,569) | 115,625 | 97.5 | $5,159 (4,869) | $2,935 (3,491) | $486 | <0.001 |
| Pharmaceuticals | 109,452 | 97.9 | $ 5,605 (8,682) | $ 1,948 (2,698) | 115,548 | 97.5 | $3,238 (6,636) | $856 (1,433) | $2,367 | <0.001 |

Supplementary Table S2: GLM analysis results: Factors associated with mean annual hospital costs (N=111,820)

| Variable | Cost ratio (CR) (95% CI) | Significance level (p-value) |
| --- | --- | --- |
| **Socio-demographic factors** | | |
| Sex |  |  |
| Female | referent |  |
| Male | 1.14 (1.13 – 1.16) | <0.001 |
| Age in years |  |  |
| 75+ | referent |  |
| 65-74 | 0.96 (0.92 – 1.00) | 0.031 |
| 50-64 | 0.89 (0.86 – 0.93) | <0.001 |
| 25-49 | 0.82 (0.78 – 0.86) | <0.001 |
| 0-24 | 1.63 (1.41 – 1.88) | <0.001 |
| Country of birth |  |  |
| Australia | referent |  |
| Other than Australia | 1.06 (1.05 – 1.08) | <0.001 |
| Marital status |  |  |
| Married / de facto | referent |  |
| Widowed/ divorced / separated | 1.17 (1.15 - 1.19) | <0.001 |
| Never married | 1.24 (1.21 – 1.27) | <0.001 |
| Occupation |  |  |
| Working | referent |  |
| Retired / not in work / home duties | 0.84 (0.61 – 1.16) | 0.287 |
| Children / students | 1.39 (1.16 – 1.66) | <0.001 |
| Accessibility and Remoteness Index of Australia (ARIA) |  |  |
| Major Cities (MC) | referent |  |
| Inner Regional (IR) | 0.98 (0.96 – 1.02) | 0.379 |
| Outer Regional (OR) | 1.06 (1.02 – 1.10) | 0.004 |
| Remote (R) | 1.36 (1.20 – 1.53) | <0.001 |
| Very Remote (VR) | 1.71 (1.53 – 1.19) | <0.001 |
| Socio-Economic Indexes for Areas (SEIFA) |  |  |
| 1 – 2 (Most Disadvantaged) | referent |  |
| 3 – 4 | 0.99 (0.97 – 1.03) | 0.963 |
| 5 – 6 | 0.96 (0.93 – 1.00) | 0.006 |
| 7 – 8 | 0.97 (0.94 – 1.01) | 0.033 |
| 9 – 10 (Most Advantaged) | 0.91 (0.88 – 0.94) | <0.001 |
| **Clinical factors** | | |
| Type of cancer, relative 5-year survival |  |  |
| Low | referent |  |
| Medium | 1.14 (1.11 – 1.18) | <0.001 |
| High | 0.89 (0.87 – 0.91) | <0.001 |
| Year of cancer diagnosis |  |  |
| 1997-2001 | referent |  |
| 2002-2006 | 0.98 (0.96 – 1.01) | 0.227 |
| 2007-2011 | 0.99 (0.97 – 1.02) | 0.413 |
| 2012-2015 | 1.35 (1.31 – 1.38) | <0.001 |
| Vital status |  |  |
| Alive in 2016 | referent |  |
| Died prior to 2016 | 1.79 (1.76 – 1.83) | <0.001 |
| **Interaction effects** |  |  |
| ARIA*SEIFA |  |  |
| ARIA very remote*SEIFA 1-2 (most disadvantaged) | referent |  |
| ARIA remote*SEIFA 5-6 | 0.82 (0.71-0.95) | 0.009 |
| ARIA remote*SEIFA 3-4 | 0.81 (0.67-0.96) | 0.012 |
| Occupation*Age |  |  |
| Age 75+*occupation ‘working’ | referent |  |
| Age 65-74*occupation ‘retired / not in work / home duties’ | 1.27 (0.91-1.75) | 0.158 |
| Age 65-74*occupation ‘children / students’ | 0.46 (0.19-1.14) | 0.095 |

Supplementary Table S3: Most common reasons for hospital treatment (MDC), related costs and length of stay (during study period 2013-2016) for N=111,820 individuals with N=682,483 hospital episodes

| Major Diagnostic Categories | N (%) | Total cost of episodes in AU$ | % of total cost | Mean episode cost in AU$ (SD) | Mean LoS in days (SD) |
| --- | --- | --- | --- | --- | --- |
| 11: Diseases and disorders of the kidney and urinary tract | 142,409 (20.9) | $354,606,614 | 7.2 | $2,490 (5,291) | 1.5 (2.5) |
| 17: Neoplastic disorders (Haematological and Solid Neoplasms) | 134,427 (19.7) | $553,779,610 | 11.3 | $4,120 (10,229) | 1.6 (3.9) |
| 6: Diseases and disorders of the digestive system | 57,840 (8.5) | $661,127,985 | 13.5 | $11,431 (17,579) | 4.0 (7.0) |
| 23: Factors influencing health status and other contacts with health services | 53,969 (7.9) | $340,284,450 | 7.0 | $6,306 (18,173) | 3.9 (12.1) |
| 5: Diseases and disorders of the circulatory system | 47,018 (6.9) | $419,226,624 | 8.6 | $8,917 (13,476) | 3.2 (5.2) |
| 4: Diseases and disorders of the respiratory system | 42,936 (6.3) | $471,341,962 | 9.6 | $10,977 (12,291) | 4.7 (6.4) |
| 1: Diseases and disorders of the nervous system | 31,763 (4.7) | $383,454,344 | 7.8 | $12,072 (17,028) | 5.4 (11.3) |
| 8: Diseases and disorders of the musculoskeletal system and connective tissue | 31,411 (4.6) | $432,904,940 | 8.8 | $13,782 (14,033) | 5.3 (8.3) |
| 9: Diseases and disorders of the skin, subcutaneous tissue and breast | 30,524 (4.5) | $248,518,777 | 5.1 | $8,142 (8,359) | 2.7 (5.3) |
| 16: Diseases and disorders of the blood and blood forming organs and immunological disorders | 21,576 (3.2) | $109,497,706 | 2.2 | $5,075 (9,757) | 2.2 (3.9) |
| 3: Diseases and disorders of the ear, nose, mouth and throat | 15,744 (2.3) | $138,512,380 | 2.8 | $8,797 (14,396) | 2.9 (5.6) |
| 7: Diseases and disorders of the hepatobiliary system and pancreas | 12,232 (1.8) | $172,911,098 | 3.5 | $14,136 (21,701) | 5.0 (6.7) |
| 18: Infectious and parasitic diseases | 11,373 (1.7) | $151,022,597 | 3.1 | $13,280 (17,870) | 5.2 (7.3) |
| 21: Injuries, poisonings and toxic effects of drugs | 10,633 (1.6) | $82,602,470 | 1.7 | $7,768 (14,366) | 3.2 (6.3) |
| 10: Endocrine, nutritional and metabolic diseases and disorders | 8,654 (1.3) | $86,804,188 | 1.8 | $10.030 (10,497) | 3.5 (5.9) |
| 12: Diseases and disorders of the male reproductive system | 8,528 (1.2) | $92,978,055 | 1.9 | $10,903 (10,792) | 3.2 (6.1) |
| 2: Diseases and disorders of the eye | 7,873 (1.2) | $35,082,438 | 0.7 | $4,456 (4791) | 1.4 (2.3) |
| 13: Diseases and disorders of the female reproductive system | 6,742 (1.0) | $78,863,015 | 1.6 | $11,698 (11,260) | 3.2 (6.6) |
| 19: Mental diseases and disorders | 4,110 (0.6) | $60,198,316 | 1.2 | $14,647 (26,219) | 8.8 (22.6) |
| 14: Pregnancy, childbirth and the puerperium | 1,555 (0.2) | $9,477,739 | 0.2 | $6,095 (6,170) | 2.1 (2.9) |
| 20: Alcohol / drug use and alcohol/drug induced organic mental disorders | 941 (0.01) | $5,572,976 | 0.1 | $5,922 (6,778) | 3.2 (4.8) |
| 22: Burns | 213 (0.0) | $2,601,770 | 0.1 | $12,216 (30,632) | 4.5 (7.6) |
| 15: Newborns and other neonates | 12 (0.0) | $324,702 | 0.0 | $27,059 (35,616) | 4.6 (5.0) |
| Total | **682,483 (100)** | **$4,891,694,760** | **100** | **$7,168 (12,929)** | **2.96 (6.7)** |

Supplementary Table S4: Overview of hospital episodes for 'MDC 17 Neoplastic disorders' AR-DRG diagnosis codes, costs and length of stay (LoS) (n=134,427), in ascending order from highest to lowest total cost

| AR-DRG code description (codes) | Treatment category | N (%) | Total cost of episodes in AU$ | % of total cost  (MDC 17) | Mean episode cost in AU$ (SD) | Mean LoS in days (SD) |
| --- | --- | --- | --- | --- | --- | --- |
| Chemotherapy (R63Z) | Medical | 111,815 (83.2) | $244,344,386 | 44.1 | $2,186 (1,857) | 1.0 (0.0) |
| Lymphoma and Non-Acute Leukaemia (R61A, R61B, R61C) | Medical | 13,832 (10.3) | $100,285,004 | 18.1 | $7,250 (11,469) | 2.9 (6.1) |
| Acute Leukaemia  (R60A, R60B, R60C) | Medical | 3,395 (2.5) | $63,675,633 | 11.5 | $18,756 (23,885) | 6.1 (10.3) |
| Bone Marrow Transplant  (A07Z, A08A, A08B) | Surgical | 697 (0.5) | $62,582,432 | 11.3 | $89,789 (70,259) | 24.9 (15.4) |
| Lymphoma and Leukaemia  (R01A, R01B, R03A, R03B) | Surgical | 1,052 (0.8) | $29,286,625 | 5.3 | $27,839 (31,470) | 8.0 (14.1) |
| Other Neoplastic Disorders / Other (R02A, R02B, R04A, R04B, A01Z) | Surgical | 1,462 (1.1) | $28,527,550 | 5.2 | $19,512 (14,422) | 4.3 (5.7) |
| Other Neoplastic Disorders / Other (R62A, R62B, A40Z) | Medical | 1,543 (1.1) | $17,768,286 | 3.2 | $11,515 (14,694) | 5.1 (7.4) |
| Tracheostomy (A06Z) | Surgical | 38 (0.0) | $5,962,108 | 1.1 | $156,897 (72,135) | 26.1 (19.5) |
| Radiotherapy (R64Z) | Medical | 593 (0.5) | $1,347,584 | 0.2 | $2,273 (869) | 1.0 (0.0) |
| Total |  | 134,427 (100) | $553,779,610 | 100 | $4,120 (10,229) | 1.6 (3.9) |

Supplementary Table S5: GLM analysis results: Factors associated with hospital episode cost (N=682,483)

| Variable | Cost Ratio (CR) (95% CI) | Significance level (p-value) | N (%) |
| --- | --- | --- | --- |
| **Socio-demographic factors** | | |  |
| Sex |  |  |  |
| Female | referent |  | 294,046 (43.1) |
| Male | 1.08 (1.07-1.08) | <0.001 | 388,437 (56.9) |
| Age in years |  |  |  |
| 75+ | referent |  | 228,372 (33.5) |
| 65-74 | 0.91 (0.89-0.93) | <0.001 | 197,949 (29.0) |
| 50-64 | 0.86 (0.84-0.87) | <0.001 | 168,476 (24.7) |
| 25-49 | 0.82 (0.80-0.84) | <0.001 | 68,396 (10.0) |
| 0-24 | 1.05 (1.00-1.11) | 0.050 | 19,290 (2.8) |
| Country of birth |  |  |  |
| Australia | referent |  | 505,223 (74) |
| Other than Australia | 1.01 (1.00 – 1.02) | 0.034 | 177,260 (26) |
| Marital status |  |  |  |
| Married / de facto | referent |  | 398,122 (58.3) |
| Widowed/ divorced / separated | 1.07 (1.06 - 1.08) | <0.001 | 199,643 (29.3) |
| Never married | 1.06 (1.04 – 1.07) | <0.001 | 84,285 (12.3) |
| Occupation |  |  |  |
| Working | referent |  | 307,992 (45.1) |
| Retired / not in work / home duties | 0.89 (0.77 – 1.03) | 0.103 | 248,047 (36.3) |
| Children / students | 0.85 (0.79 – 0.92) | <0.001 | 16,152 (2.4) |
| Accessibility and Remoteness Index of Australia (ARIA) |  |  |  |
| Major Cities (MC) | referent |  | 370,620 (54.3) |
| Inner Regional (IR) | 0.90 (0.89 – 0.91) | <0.001 | 183,346 (26.9) |
| Outer Regional (OR) | 1.06 (1.04 – 1.08) | <0.001 | 111,797 (16.4) |
| Remote (R) | 0.85 (0.82 – 0.89) | <0.001 | 10,405 (0.9) |
| Very Remote (VR) | 2.10 (2.00 – 2.21) | <0.001 | 5,838 (0.9) |
| Socio-Economic Indexes for Areas (SEIFA) |  |  |  |
| 1 – 2 (Most Disadvantaged) | referent |  | 197,285 (28.9) |
| 3 – 4 | 1.10 (1.08 – 1.11) | <0.001 | 152,779 (22.4) |
| 5 – 6 | 1.01 (1.00 – 1.03) | 0.084 | 148,304 (21.7) |
| 7 – 8 | 1.06 (1.05 – 1.08) | <0.001 | 114,873 (16.8) |
| 9 – 10 (Most Advantaged) | 1.07 (1.06 – 1.09) | <0.001 | 68,740 (10.1) |
| **Clinical factors** | | |  |
| Type of cancer, relative 5-year survival |  |  |  |
| Low | referent |  | 84,876 (12.4) |
| Medium | 0.89 (0.88 – 0.90) | <0.001 | 109,254 (16.0) |
| High | 1.04 (1.03 – 1.05) | <0.001 | 420,905 (61.7) |
| Time since diagnosis in years |  |  |  |
| 15-20 | referent |  | 46,840 (6.9) |
| 10-14 | 1.02 (1.01 – 1.04) | 0.002 | 95,355 (14.0) |
| 5-9 | 1.04 (1.02 – 1.05) | <0.001 | 155,098 (22.7) |
| 2-4 | 1.09 (1.07 – 1.10) | <0.001 | 156,435 (22.9) |
| 0-1 | 1.38 (1.37 – 1.40) | <0.001 | 228,755 (33.5) |
| Vital status |  |  |  |
| Alive | referent |  | 662,788 (97.1) |
| Died during hospital episode | 1.61 (1.58 – 1.64) | <0.001 | 19,695 (2.9) |
| Care type |  |  |  |
| Acute | referent |  | 641,799 (94.0) |
| Other | 3.91 (3.81– 4.01) | <0.001 | 10,270 (1.5) |
| Palliative care | 2.34 (2.29 – 2.38) | <0.001 | 18,731 (2.7) |
| Rehabilitation care | 2.94 (2.87 – 3.01) | <0.001 | 11,683 (1.7) |
| **Interaction effects** |  |  |  |
| ARIA*SEIFA |  |  |  |
| ARIA very remote*SEIFA 1-2 (most disadvantaged) | referent |  | - |
| ARIA remote*SEIFA 5-6 | 1.43 (1.35-1.51) | <0.001 | - |
| ARIA remote*SEIFA 3-4 | 1.67 (1.56-1.79) | <0.001 | - |
| Occupation*Age |  |  |  |
| Age 75+*occupation ‘working’ | referent |  | - |
| Age 65-74*occupation ‘retired / not in work / home duties’ | 1.14 (0.99-1.32) | 0.073 | - |
| Age 65-74*occupation ‘children / students’ | 1.86 (1.25-2.77) | 0.002 | - |
